# Analysis of the Stress Field in Photoelasticity Used to Evaluate the Residual Stresses of a Plastic Injection-Molded Part

**DOI:** 10.3390/polym15163377

**Published:** 2023-08-11

**Authors:** Carlos Vargas-Isaza, Juan Posada-Correa, Juan Briñez-de León

**Affiliations:** 1 Grupo Investigación Materiales Avanzados y Energía, Instituto Tecnológico Metropolitano, Medellín 050034, Colombia; 2 Grupo Investigación Calidad, Metrología y Producción, Instituto Tecnológico Metropolitano, Medellín 050034, Colombia; 3 Grupo de Investigación GIIAM, Institución Universitaria Pascual Bravo, Medellín 050034, Colombia

**Keywords:** photoelasticity, residual stress, injection molding, mechanical properties, color demodulation, decoding of color

## Abstract

The degree of quality of thermoplastic injection-molded parts can be established based on their weight, appearance, and defects. However, the conditions of the injection process may induce effects on the mechanical performance of the injected parts, and the residual stresses can cause cracks or early failures when an external load or force is applied. To evaluate these mechanical behaviors, different experimental techniques have been reported in the literature, where digital photoelasticity has stood out both for being a non-contact technique and for achieving quantitative results through sophisticated computational algorithms. Against this background, our proposal consists of analyzing the overall residual stress distribution of parts injected under different molding conditions by using digital photoelasticity. In this case, the specimens are subjected to bending strength tests to identify possible effects of the injection process conditions. The findings show that, at mold temperatures of 80 °C, flow-induced residual stresses increase with packing pressure. However, these internal stress levels do not affect the external load applied by the mechanical bending test, while the mass injected at higher levels of packing pressure helps to increase the bending strength of the injected part. At lower mold temperatures (50 °C), the mechanical strength of the injected part is slightly reduced, possibly due to a lower effect of the packing pressure.

## 1. Introduction

Digital photoelasticity is an experimental technique used to evaluate the stress distributed in a body subjected to loading conditions. This technique has become relevant alongside technological development due to the possibilities it offers, including non-destructive experiments, non-invasive evaluation, and computational support. In principle, digital photoelasticity takes advantage of the fact that birefringent bodies can reveal stress information through color fringe patterns; therefore, it is necessary to demodulate the information in the images to quantify stress. In this field of study, new computational developments have favored the transition from conventional techniques based on manual quantification of color fringe patterns to computational technologies based on image processing by means of polarization states, quantification of light intensity [1,2], and determination of phase delay through unwrapping strategies [2,3,4,5]. Such analyses have been performed on bodies made of different types of birefringent materials; notwithstanding, translucent polymers have been more popular because their optical properties have been deeply studied.

Although different types of thermoplastic materials have been used in the engineering field, polycarbonate stands out for being widely employed in optical, optoelectronic, and electronic applications. However, residual stresses have a negative effect on the optical properties required for these developments [6,7], as well as on the expected mechanical performance of plastic parts [8,9]. For this reason, numerous studies have been published on the reduction of residual stresses in polycarbonate parts [1,10,11]. In this sense, computer simulations have contributed to the analysis and prediction of residual stresses in injected parts. However, this may require simplifications in the calculation models or, in the case of small parts, calculations may differ from the real residual stresses caused by the injection process, so directly measuring the product is also necessary. In response to these needs, the literature on digital photoelasticity includes studies that analyze residual stresses in polycarbonate specimens, making it an easy-to-implement quality control technique. Specifically, photoelasticity has made it possible to assess residual stress information by unwrapping fringes in images [10].

In the case of polycarbonate, photoelasticity studies have focused on the birefringence phenomenon that occurs when this material is subjected to mechanical stress conditions, allowing stress information to be revealed through color fringe patterns that must subsequently be decoded using computational strategies. In this regard, the literature describes conventional methods that include phase shifting [12], load stepping [13], color demodulation (Twelve Fringe Photoelasticity, TFP) [1], and regularized phase-tracking techniques [14], among others. In those cases, common findings indicate that difficulties could be related to making fast evaluations, given the algorithm requirements due to the complicated optical setups in the image acquisition process, induction of dynamic states, heavy optimization methods, and sensitivity to different stress values with similar color representations. In addition, the recently developed PhotoelastNet network addresses some of these difficulties, but the trained model is not yet available for open access [15].

Furthermore, as observed in the literature about residual stresses, studies on digital photoelasticity report advantages over other experimental methods [16]. The latter need to induce dynamic load states to observe the mechanical phenomenon, while the former can visualize the stresses by simply exposing the object to the optical light polarization system. Considering these advantages, we focus on digital photoelasticity to analyze the residual stresses and mechanical performance of a polycarbonate injected part fabricated under different injection conditions and subsequently subjected to mechanical bending tests. In this study, residual stress is quantified using the TFP technique, which is easier to implement once the different stress representations in the color fringe patters are known and organized as a reference color chart. Here, the color chart comes from an experimental identification of 50 points in the stressed specimens, ensuring their distribution between the minimum and maximum possible stress values. Within the process, we initially express the stress values in fringe order and subsequently convert them into mechanical units using the stress-optical law.

## 2. Materials and Methods

### 2.1. Injection of Plastic Parts

**Figure 1:**
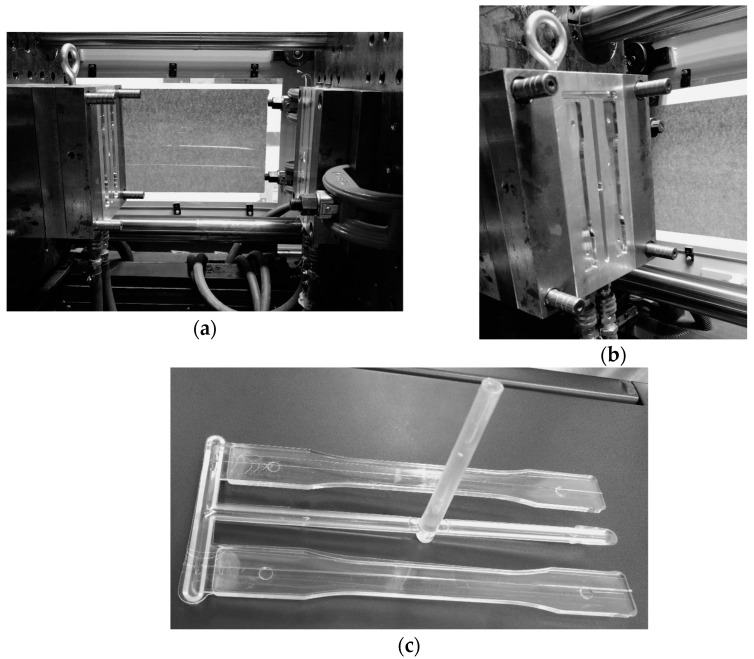
Mold used for the injection of the specimens to be analyzed (**a**), detail of cavity (**b**), and injected plastic part (**c**).

Polycarbonate (LEXAN 144R), a type of amorphous plastic supplied by General Electric Plastics, was injected into the specimen mold (see Figure 1) under different molding and packing conditions (see Table 1). The cooling time in the mold after the injection stage (cavity filling and packing) was set at 15 s. This time was determined by analytically calculating the total cooling time of a rectangular section using Equation (1), considering the injection time and the remaining cooling time [17,18]. The thermal properties of the injected polycarbonate were obtained from literature data and the melting (305 °C), mold cavity (50 °C and 80 °C), and demolding (100 °C) temperatures were taken from the recommended ranges of the plastic manufacturer’s datasheet. All the specimens obtained in this study were injected using a WELLTEC TTI-90F2V horizontal injection molding machine from Welltec Machinery Limited in Hong Kong, China, with a 90-ton clamping force to carry out the subsequent photoelasticity and bending tests.

(1) $$t_{enfT}=\left(\frac{t^{2}}{pi^{2}\times Dif}\right)\times Ln\left(\frac{8\times \left(Tm-Tc\right)}{pi^{2}\times \left(Te-Tc\right)}\right)$$

where:

*t_enfT_*: total cooling time [s].
*t*: injected part thickness (3.2 mm).
*pi*: number π.
*Dif*: effective thermal diffusivity of the material [mm^2^/s].
*Tm*: average melting temperature [°C].
*Tc*: average mold cavity temperature [°C].
*Te*: average ejection temperature of the injected part [°C].

**Table 1:** Experimental design of the injection process conditions using an injection temperature of 305 °C, a filling time of 1 s, and a remaining cooling time of 15 s.

Packing Time [s]	Packing Pressure [Bar]	Mold Temperature [°C]
4	40	50
7.7	40	50
4	60	50
7.7	60	50
4	40	80
7.7	40	80
4	60	80
7.7	60	80

### 2.2. Photoelasticity Testing and Residual Stress Calculation

**Figure 2:**
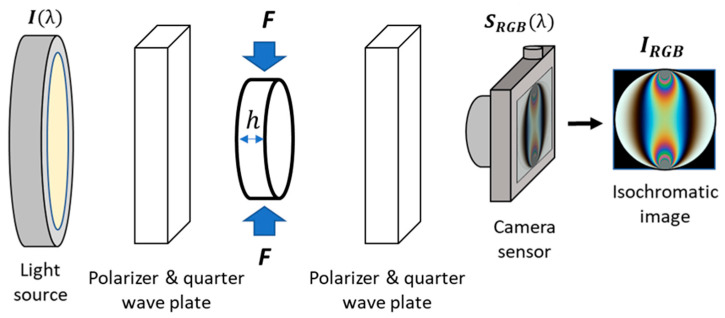
Schematic of a circular polariscope implemented around a disk under compression.

In digital photoelasticity studies, the birefringence of a translucent body can modify the phase of light traveling through it. Such phase delay ‘δ’ is proportional to the optical properties of the material ‘C’, the thickness ‘h’, the difference of the principal stresses ‘$\sigma_{1}-\sigma_{2}$’, and the wavelength of the light radiation used for the visualization of the phenomenon ‘λ’. This relationship is known in the literature as the stress-optical law Equation (2) and indicates that, for controlled experimental scenarios, stresses could be quantified as a problem whose objective is to measure the phase delay [19]. In those cases, visualizing the phase delay is possible through images with color fringe patterns when the loaded specimen is observed in an optical setup called polariscope, as shown in Figure 2.

(2) $$\delta =\frac{2\pi hC\left(\sigma_{1}-\sigma_{2}\right)}{\lambda }$$

In the polariscope, the distribution of emergent light intensities ‘$I_{RGB}$’ becomes a modulation function associated with the light source, the configuration of the polarizers, the phase delay related to the stress-optical law, and the spectral response of the camera [20]. The literature describes several polariscope models and setups, where any movement of the optical elements could affect the resulting intensities and, therefore, the fringe pattern representations. Consequently, the circular configuration is often used experimentally due to its optical simplicity and the fact that the resulting fringes are not affected by the principal stress directions—an effect known as isoclinic fringes. In this study, we adopted the circular configuration, where the Jones calculus can be used to model the emergent intensities, as shown in Equation (3).

(3) $$I_{RGB}=\frac{1}{2}\left(1+\cos\delta \right)$$

After the stress distribution has been visualized using color fringe pattern images, a demodulation stage is carried out to quantify the stress measurements. In this case, we followed the TFP method. Here, the computational principle consists in search strategies where, for a reference part, each color emerging in the fringes is stored with its respective stress value in a reference color chart called Lookup Table (LUT) [15]. Thus, to decode a new experimental case, each color must be looked up in the LUT. Although the literature on photoelasticity reports several search strategies for TFP, the most common is the Euclidian distance because of its smooth calculation, as shown in Equation (4). There, $C1_{RGB}$ represents each color in the target image and $C2_{RGB}$ accounts for each color in the LUT, both expressed in the RGB color space. In addition, $C1_{R}$, $C1_{G}$, and $C1_{B}$ are all RGB components of the target color. Likewise, $C2_{R}$, $C2_{G}$, and $C2_{B}$ are the RGB components of the reference colors integrating the LUT.

(4) $$d\left(C1_{RGB},C2_{RGB}\right)=\sqrt[{2}]{{\left(C1_{R}-C2_{R}\right)}^{2}+{\left(C1_{G}-C2_{G}\right)}^{2}+{\left(C1_{B}-C2_{B}\right)}^{2}}$$

**Figure 3:**
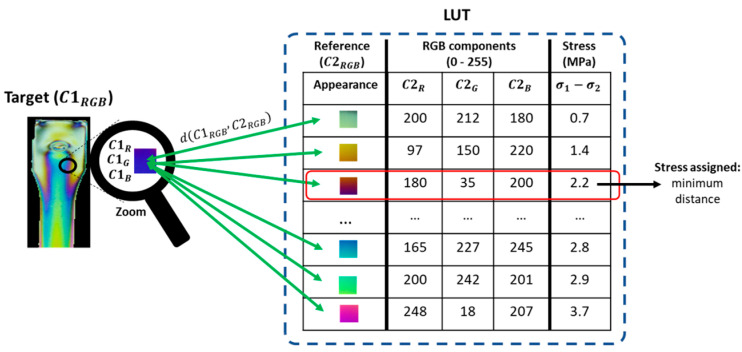
Computational decoding strategy in photoelasticity to obtain quantifiable stress measurements.

As a graphical representation of the color demodulation process, Figure 3 summarizes the steps required for demodulating the stress information wrapped by color fringe patterns in a photoelasticity image. There, the algorithm takes as input, on the one hand, the LUT previously created with a reference sample and, on the other hand, the new experimental image that needs to be demodulated, ‘$C1_{RGB}$’. Then, each color in the image is compared with each color stored in the LUT using the Euclidean distance. Subsequently, the stress value is assigned taking into account the reference value in which the minimum distance was obtained [1].

**Figure 4:**
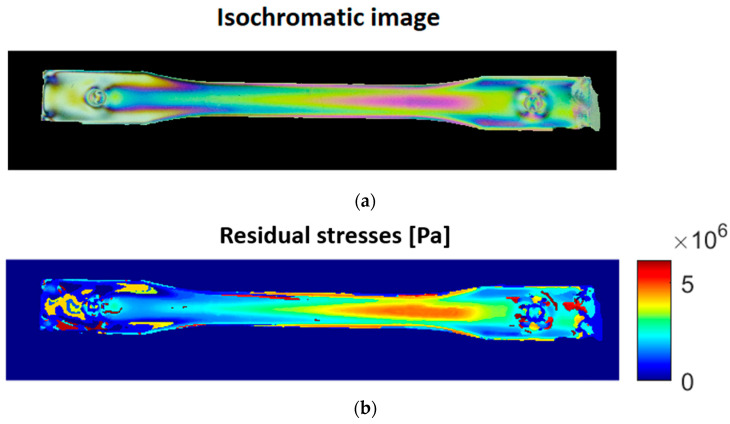
Image processing obtained by photoelasticity. (**a**) Isochromatic image obtained through the experimental setup; (**b**) image of the stress map demodulated from the previous image.

When all the pixels in the target image have been searched in the LUT, the photoelasticity image (Figure 4a) is transformed into a stress map (Figure 4b), which corresponds to the residual stresses present in the injected part. Following this procedure and aiming to verify the repeatability of the residual stress pattern, the photoelasticity calculation procedure is performed on three injected parts for each condition listed in Table 1. In addition, the residual stress distribution of polycarbonate is calculated using an average value of the optical stress coefficient, which depends on the glass transition temperature and the polycarbonate modulus [11,21].

### 2.3. Mechanical Bending Tests

The rectangular area of the injected part was subjected to bending tests according to ASTM D790—Method A: Flexural strength to evaluate the application of the plastic part under more realistic loading conditions.

The dimensions of the specimens used were depth 3 mm, width 12.7 mm. The tests were performed on a on a Shimadzu/AG 100 kNX universal testing machine, from manufacturer Shimadzu Corporation in Kioto, Japan, with a 10-kN load cell. Five replicates of each injection condition were evaluated to obtain the mean and standard deviation.

## 3. Results

### 3.1. Photoelasticity

**Figure 5:**
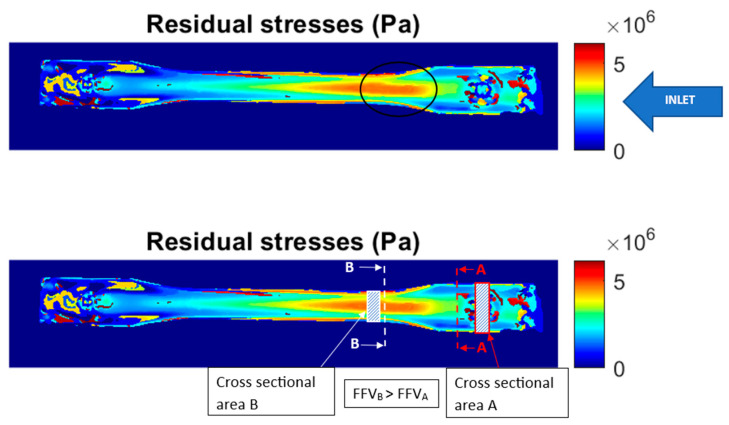
Distribution of principal stresses in the injected part (residual stresses).

This analysis was based on the principal stress difference distribution image. Figure 5 shows the distribution of residual stresses in the injected part. A gradual increase in the stresses can be observed from the inlet of the mold cavity to the area where the width of the injected specimen is narrowed. This can be explained by the fact that the injected plastic flow front velocity (FFV) is accelerated in this area of the mold cavity, as the cross-sectional area is reduced using Equation (4).

(5) $$FFV=\left(\frac{\dot{V}}{Cross-sectional\;area}\right)=\left(\frac{\dot{V}}{\left(t\times w\right)}\right)$$

where:

*FFV*: Flow front velocity [mm/s]
$\dot{V}$: Volumetric flow rate of melted plastic [mm^3^/s]
*t*: part thickness [mm]
*W*: part width [mm]

This difference in the FFV leads to multiple molecular orientations during the flow of the melted plastic, which generate molecular relaxation during the cooling and solidification process, ultimately promoting different levels of residual stresses. These flow-induced residual stresses have been widely discussed in previous studies on injection-molded parts [22,23,24,25,26]. Consequently, we needed to determine whether the other mechanism of formation of thermally induced residual stresses (which are the result of non-uniform cooling of the melted polymer) had a greater or lesser influence on the case analyzed in this study. To this end, we simulated the injection process of the part under analysis using Moldex 3D R12 software and reproduced the same process conditions, geometry of the injected part, and mold configuration (feeding and cooling channels).

**Figure 6:**
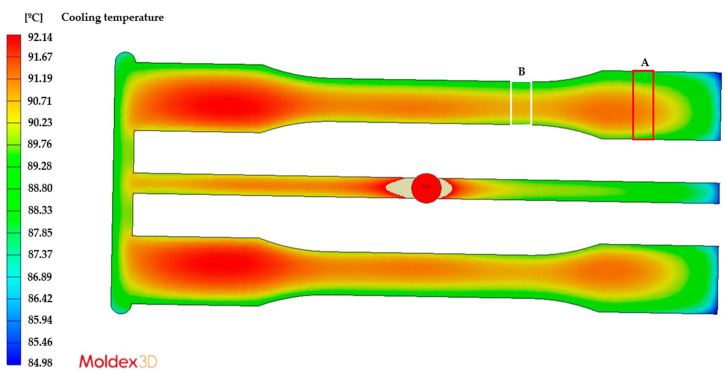
Temperature distribution of the injected part at a mold temperature of 50 °C.

**Figure 7:**
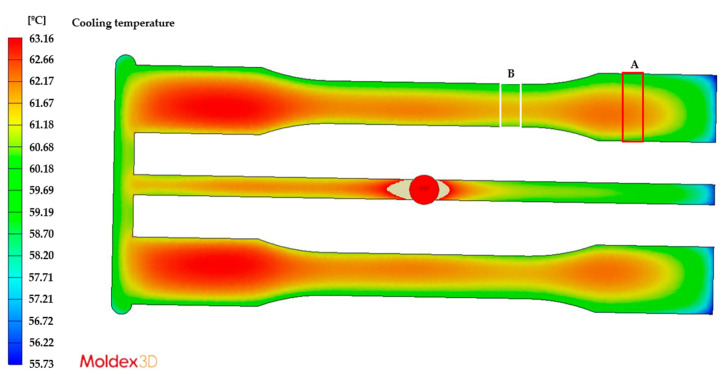
Temperature distribution of the injected part at a mold temperature of 80 °C.

Figure 6 and Figure 7 show the temperature distribution of the injected part for the cooling time established in the analyses, with a mold cooled with cooling liquid at 50 °C and 80 °C, respectively. In both cases, we observed that the highest temperature gradients occurred between both ends of the part, which are equivalent to the residual stress levels shown in Figure 5. However, when comparing Areas A and B in Figure 5, Figure 6 and Figure 7, the temperature differences are a maximum of 1 °C, both for the 50 °C and the 80 °C mold, which indicates a uniform cooling process between these two areas. In other words, the overall cooling of the mold is uniform except for the ends. Therefore, we could say that the mechanism of residual stress formation in this part is mainly flow-induced, which is more evident between Areas A and B in Figure 5.

Similarly, a recent study by Chen and collaborators evaluated the residual stresses in injected polycarbonate parts of different thicknesses. The authors found that, for thicknesses of 3 mm, the residual stresses are due exclusively to the effect of flow, which is in agreement with the results of the present study [27].

#### 3.1.1. Effect of the Mold Temperature

**Figure 8:**
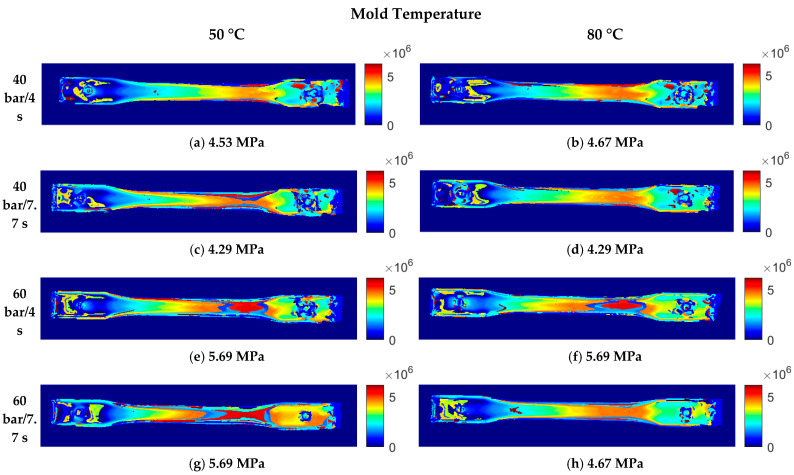
Residual stress distribution in a polycarbonate injection-molded part under different process conditions.

Figure 8 shows the summary of the residual stress distributions for each set of injection conditions listed in Table 1. It also presents residual stress values in the narrower area of the injected part, as shown in Figure 5 (Area B). Although the values do not show a very significant difference, we can observe greater areas with high residual stresses (red areas) in the parts injected using the 50 °C mold, which indicates higher residual stress levels. This behavior is due to the fact that, at lower temperatures, there is a faster solidification of the injected material and a higher degree of molecular orientation, which leads to an increase in the residual stress. We also observed that the color transition (stress levels) is more gradual in the 80 °C mold than in the 50 °C mold, which may be related to the solidification speed of the injected material.

#### 3.1.2. Effect of the Packing Pressure

This variable has a stronger effect on the residual stress levels. In Figure 8, for the mold temperature of 50 °C and the packing time of 4 s, we observed that, when increasing the packing pressure from 40 to 60 bar, the color of the residual stress distribution intensifies in the narrower area of the injected part. It corresponds to a variation of 4.53 MPa to 5.69 MPa, considering the selected control point (Figure 8a,e). The same occurred when comparing the residual stresses at the packing time of 7.7 s, where the residual stress increased from 4.29 MPa to 5.59 MPa at the selected control point (Figure 8c,g).

We observed the same trend for the mold temperature of 80 °C (Figure 8b vs. Figure 8f and Figure 8d vs. Figure 8h). The increase in the packing pressure intensifies the molecular orientation of the injected plastic, generating higher residual stress levels. Since the molecular orientations are different in the mold-filling and packing stages, stress distributions through the thickness of the part are established during the cooling stage, with tension in the solidified layer and compression in the core of the specimen [28]. Therefore, increasing the packing pressure also increases the residual stress levels (tension and compression) through the thickness of the part. This behavior has been confirmed by other studies on residual stresses in injection-molded parts [26,29].

#### 3.1.3. Effect of the Packing Time

A similar analysis can be conducted by comparing the packing times. However, in this case, when the time is increased, the residual stress is slightly reduced, as observed when comparing the conditions of 40 bar/4 s and 40 bar/7.7 s for a mold temperature of 50 °C (Figure 8a,c). The same happens when comparing 40 bar/4 s and 40 bar/7.7 s, as well as 60 bar/4 s and 60 bar/7.7 s (Figure 8b,d,f,h) for a mold temperature of 80 °C. By contrast, there were no variations when comparing 60 bar/4 s and 60 bar/7.7 s for a mold temperature of 50 °C (Figure 8e,g).

Nevertheless, the trend seems to show a reduction in residual stress levels, which is supported by similar studies. The packing time affects both the material shrinkage and molecular relaxation, considering that the filling, packing, and remaining cooling times make up the total cooling time in the mold. In this sense, there is no consensus regarding the effect of the packing time, because it may depend on the interaction with other process variables [30,31].

#### 3.1.4. Analysis of the Interaction of the Process Variables

According to the above discussion, we cannot determine the direct effect of each process variable on the residual stresses because multiple factors occur and compete during the injection process. Such factors include: the cooling rate of the material induced by the mold temperature; the internal pressure of the material in the cavity after the packing stage; and the cooling time of the injected part in the mold, which gives way to the differentiated molecular relaxation and the contraction of the material. Based on this approach and the data obtained from the photoelasticity images, the effect of the input variables (mold temperature and packing pressure and time) on the response of the residual stress value was analyzed. This is statistically represented in a 3^2^ factorial analysis of cube plots, principal effects, variable interactions, and Pareto diagrams, which are presented and analyzed below.

**Figure 9:**
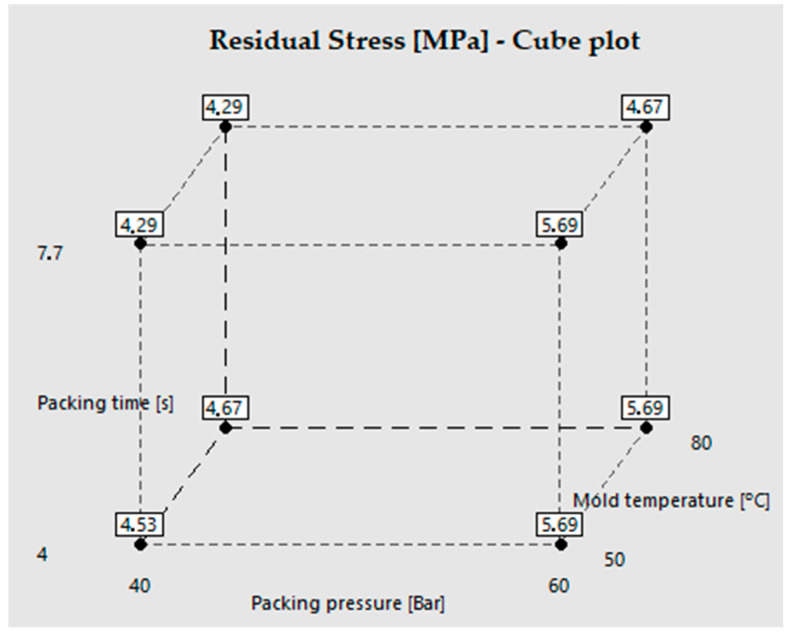
Cube plot of the residual stresses in the narrower area of the injected part.

Figure 9 shows a cube plot that summarizes the variation of the average residual stress values with respect to the experimental photoelasticity results. Each point corresponds to one of the process conditions under analysis.

**Figure 10:**
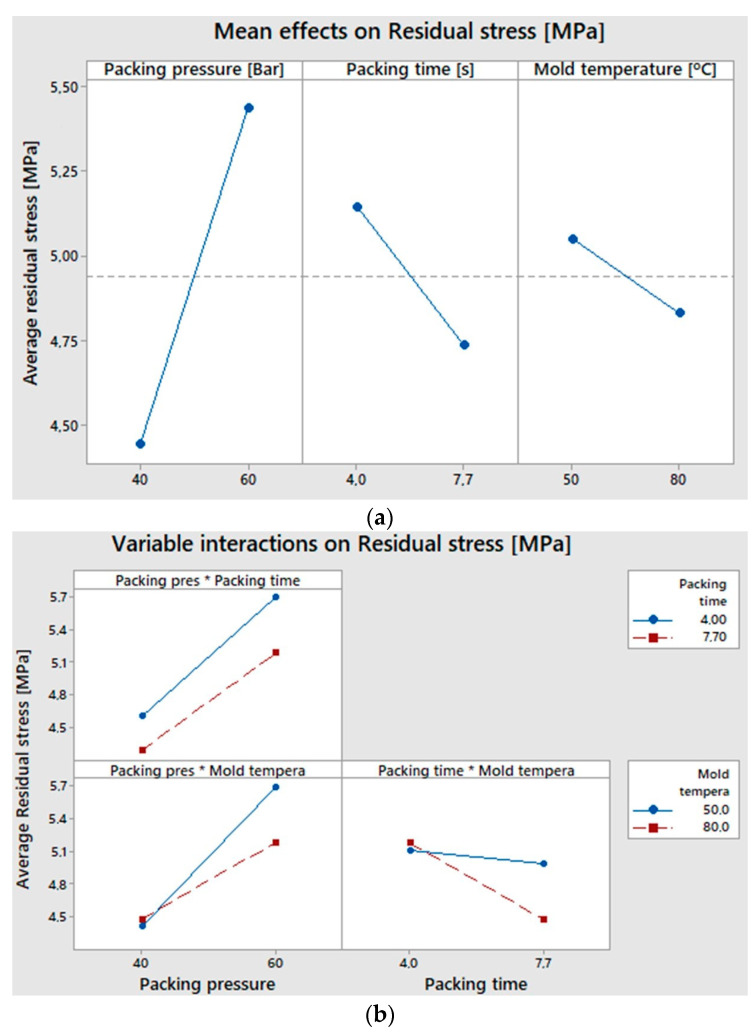

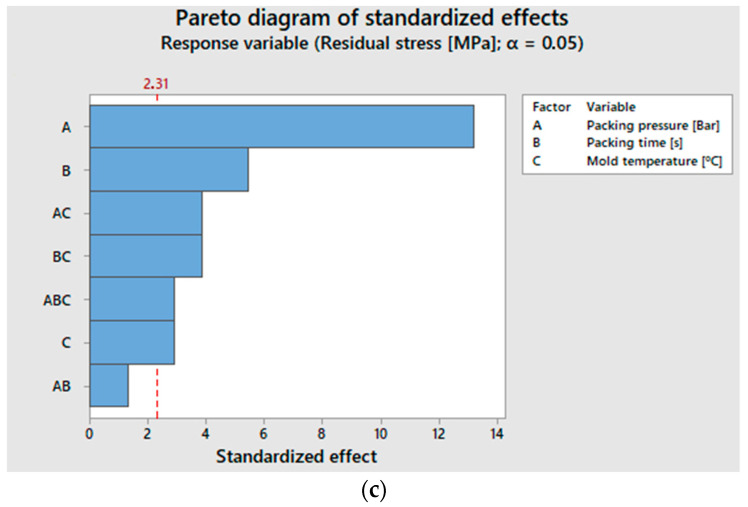
Representation of the effect of the process variables on the residual stress based on a statistical 32 factorial analysis. (**a**) Principal effects; (**b**) variable interactions; and (**c**) Pareto diagram.

Figure 10 details the effect of the process variables on the residual stresses generated in the injected part. As previously discussed, Figure 10a indicates that the variables with the greatest impact on residual stresses are, in order, packing pressure, packing time, and mold temperature. It is also worth mentioning the interaction between process variables and their effect on residual stress (Figure 10b). Regarding this, we found that the combination of the lowest level of packing pressure (40 bar) with the highest level of packing time (7.7 s) produced the lowest residual stress (4.29 MPa), regardless of the mold temperature. This result could suggest that a longer cooling time in the mold (7.7 s packing + 15 s cooling) favors a higher molecular relaxation, without inducing high molecular orientations by employing a lower packing pressure (40 bar). The interaction of the packing pressure with the mold temperature suggests that, when using the lowest packing pressure level (40 bar), the residual stresses are very similar at mold temperatures of both 50 °C and 80 °C (4.41 MPa and 4.48 MPa); therefore, the impact of the packing pressure is higher.

Finally, the interaction of a packing time of 7.7 s and a mold temperature of 80 °C resulted in the lowest residual stress because it favored a lower cooling rate and a greater molecular relaxation. This last interaction is also validated in a previous study by Poszwa and collaborators, who highlighted the importance of the packing time in the injection process and its effect on residual stress [31].

Lastly, Figure 10c shows the variables and interactions that have greater weight in the generation of residual stress. They are, from the highest to the lowest, the packing pressure, the packing time, the packing pressure/mold temperature interaction, and the packing time/mold temperature interaction. The principal effect of the mold temperature has a lower weight, which could be explained by the uniformity of the mold temperature, despite the 30 °C difference between the two temperature levels.

### 3.2. Mechanical Bending Tests

**Figure 11:**
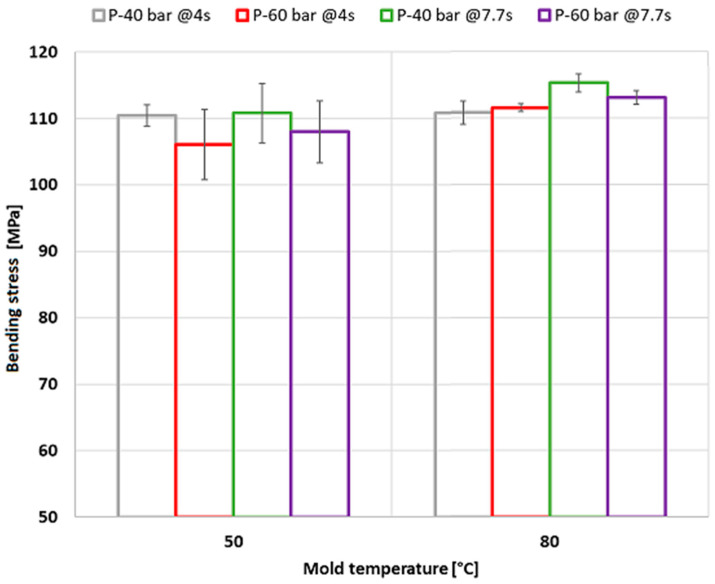
Bending stress of polycarbonate injection-molded parts.

The entire experimental design was subjected to bending tests. Figure 11 presents the summary of results. Regarding the mold temperature, we observed a slightly higher bending strength at the higher mold temperature used (80 °C), which is closer to the temperature recommended for the material [32]. This is explained by the fact that the hotter the mold is, the slower the cooling rate, which favors a more effective application of the packing stage and translates into higher weight and density and less material shrinkage. Likewise, this condition leads to a greater molecular relaxation in the injected material, which reduces the internal or residual stresses that can affect the external load applied during the bending test. Furthermore, applying a greater packing increases the weight and reduces the shrinkage of the injected product, thus improving its mechanical performance. Similar studies also describe a favorable effect of the packing stage on mechanical properties [33,34]. Finally, increasing the cooling time did not seem to significantly influence the mechanical strength of the injected product.

By analyzing the residual stresses and mechanical strength of the injected part, we found that the levels obtained under the different process conditions vary between 4.29 MPa and 5.69 MPa. These levels do not seem to be critical, considering the maximum stress that the injected part can withstand. However, some conditions of the injection process can increase the mechanical strength and prevent residual stress from leading to premature failures of the specimen subjected to external loads, as in this case of bending. For example, a temperature of 80 °C is adequate and agrees with the recommendations of the supplier of the material to be injected. The processing times can also be conveniently adjusted for a more productive process, as well as the packing pressures, provided that they do not require high technical specifications for the injection-molding equipment.

## 4. Conclusions

Using the digital photoelasticity study and a hybrid approach based on the TFP color demodulation algorithm, we obtained a residual stress distribution or map of the injected part that allowed us to identify and understand the most critical areas. Unlike other experimental techniques for residual stress estimation, the effectiveness of our proposal supported by digital photoelasticity lies in its non-invasive and non-destructive effect, which could be explored in future studies for real-time inspections in a polycarbonate production line. This inference derives from the fact that, with the reported results, we managed to determine the effect of each injection process condition on the distribution of residual stresses. Moreover, the computation algorithm enabled us to rapidly and automatically extract certain reference points from which to track the stresses generated in the injected part.

Likewise, we could determine and demonstrate that the prevailing residual stresses for the injected part and the process conditions evaluated are those induced by the flow. The temperature gradients experienced by the injected part during cooling are low; therefore, the residual stress due to temperature is minimal.

The analysis of the process variables revealed that the variables with the greatest impact on the residual stresses are, respectively, packing pressure and packing time. Mold temperature does not have a direct effect, but it does have a significant effect when in interaction with the variables during the packing stage (pressure and time).

Lastly, the residual stress levels obtained are much lower than the stresses observed in the bending tests of the injected part, so there is no detriment to the mechanical performance when applying the external bending load. This means great flexibility in the process conditions, which can be translated into enhanced productivity conditions or in the use of injection equipment that does not require high injection pressures.

## Data Availability

Not applicable.
